# Divergent Climate Sensitivity and Spatiotemporal Instability in Radial Growth of Natural and Planted *Pinus tabulaeformis* Forests Across a Latitudinal Gradient

**DOI:** 10.3390/plants14101441

**Published:** 2025-05-12

**Authors:** Yue Fan, Yujian Zhang, Dongqing Han, Yanbo Fan, Yanhong Liu

**Affiliations:** 1 School of Ecology and Nature Conservation, Beijing Forestry University, Beijing 100083, China; yyfan@bjfu.edu.cn (Y.F.); kotenbu@163.com (Y.Z.); handongqing1026@163.com (D.H.); 2 School of Geographical Sciences, Shanxi Normal University, Taiyuan 030031, China; 15203489882@163.com; 3 Beijing Key Laboratory of Forest Resources and Ecosystem Process, Beijing Forestry University, Beijing 100083, China

**Keywords:** natural and planted forests, climate change, growth response, latitudinal gradient

## Abstract

A deeper understanding of growth–climate relationships in natural forests (NFs) and planted forests (PFs) is crucial for the prediction of climate change impacts on forest productivity. Yet, the mechanisms and divergences in climatic responses between these forest types remain debated. This study investigated *P. tabulaeformis* NFs and PFs in China using tree-ring chronologies to analyze their radial growth responses to climatic factors and associated temporal–spatial dynamics. The results reveal significant negative correlations between radial growth and mean temperatures (Tmean) in August of the previous year and June of the current year, and positive correlations were observed with the September standardized precipitation evapotranspiration index (SPEI) of the previous year and May precipitation (PPT) and SPEI of the current year. Compared with NFs, PFs exhibited a heightened climatic sensitivity, with stronger inhibitory effects from prior- and current-year growing-season temperatures and greater SPEI influences during the growing season. Moving window analysis demonstrated higher temporal variability and more frequent short-term correlation shifts in PF growth–climate relationships. Spatially, NFs displayed latitudinal divergence, autumn Tmean shifted from growth-suppressive in southern regions to growth-promotive in the north, and winter SPEI transitioned from positive to negative correlations along the same gradient. However, PFs showed no significant spatial patterns. Relative importance analysis highlighted water availability (PPT and SPEI) as the dominant driver of NF growth, whereas temperature, moisture, and solar radiation co-regulated PF growth. These findings provide critical insights into climate-driven growth divergences between forest types and offer scientific support for the optimization of NF conservation and PF management under accelerating climate change.

## 1. Introduction

Forests serve as a vital component of Earth’s terrestrial ecosystems, and they play crucial roles in maintaining species distribution, biodiversity, forest productivity, and the stability of the global carbon cycle [1,2,3]. However, ongoing climate change has considerably increased the complexity and instability of forest ecosystem processes [4,5]. According to the IPCC Sixth Assessment Report, the global average surface temperature increased by approximately 1.1 °C from the pre-industrial period (1850–1900) to 2011–2020. It is expected that over the next 20 years (2021–2040), global temperatures will rise to or exceed 1.5 °C above pre-industrial levels. Intensified climate warming has already increased the frequency and intensity of extreme weather events. This trend will negatively affect forest productivity, subsequently altering forest structure and species composition, and profoundly impacting ecosystem services [6,7]. Therefore, in the context of global warming, the relationship between tree growth and climatic factors must be investigated to assess the adaptive capacity of forest ecosystems and formulate effective management strategies [8,9].

Tree rings, with their well-established chronology, continuous record, and precise temporal resolution, have been widely used to quantitatively assess the effects of climatic factors on tree radial growth [10,11,12]. However, existing studies predominantly focus on single forest types (e.g., natural forests (NFs) or planted forests (PFs)), and comparative research on forests of different origins remains contentious [13,14,15,16]. Some scholars argue that NFs, with their complex community structures and genetic diversity, may buffer climate change pressures through niche complementarity effects [17], whereas PFs, given their homogeneous planting and high-density management, can exhibit a greater climate sensitivity [18,19]. Conversely, intensive management practices in PFs (e.g., irrigation and thinning) may reduce climate dependency, which results in a lower sensitivity compared with NFs [20,21]. The relationship between NFs and PFs in response to climate change remains debated, with most research concentrated on specific regions or localized spatial scales [16,20], which leaves a critical knowledge gap in the comparison of their response differences across varying spatial scales.

A growing number of studies have explored the spatial responses of tree growth to climatic variability, and the results reveal that some species exhibit clinal variation in growth–climate relationships along environmental gradients, such as latitude or moisture availability [22,23]. Li et al. [24] demonstrated that the moisture sensitivity of Pinus sylvestris growth in Liaoning Province, China, significantly decreased along a west-to-east (north-to-south) moisture gradient. Similarly, Lyu et al. [25] discovered that as latitude decreases in Northeastern China, the relationship between *Pinus koraiensis* growth and monthly precipitation shifted from negative to positive correlations, and its positive correlation with monthly temperature gradually weakened. Notably, these spatial differentiation patterns may exhibit substantial divergence among forests of different origins. Fundamental differences between NFs and PFs in soil nutrient cycling, community structure, and microclimate regulation can lead to distinct climate response mechanisms along environmental gradients [26,27]. According to Ni et al. [28], drought-limiting effects of *Pinus massoniana* PFs intensified with the increase in latitude from south to north, whereas no such pattern was detected in NFs. These findings underscore the critical need for targeted studies that compare climate change-induced differences in radial growth responses between NFs and PFs across broader regional scales.

Temperatures in Northern China have increased significantly and have consistently remained above the national average, showing a clear warming and drying trend [29]. *Pinus tabuliformis*, endemic to China, is a key species for ecological restoration in Northern China. Its annual growth rings are clearly defined, making them a reliable record of climate and environmental changes [30,31,32]. As a critical proxy for the investigation of climate change, *P. tabulaeformis* tree rings have been extensively utilized in dendroecological research [33,34]. In this work, we analyzed the growth patterns and growth–climate relationships of NFs and PFs along a latitudinal gradient to predict the potential climate change impacts on *P. tabulaeformis* growth. We addressed the following scientific questions: (1) What are the primary climatic drivers of radial growth in NFs and PFs, and do their growth–climate relationships exhibit temporal stability? (2) Do the growth–climate relationships of *P. tabulaeformis* demonstrate spatial heterogeneity along the latitudinal gradient? The findings will reveal divergence in climate adaptation strategies between forests of differing origins and provide theoretical foundations for differentiated forest management and climate-adaptive practices.

## 2. Results

### 2.1. Chronology Statistical Characteristics

Based on the statistical characteristics of the standard chronologies of *P. tabulaeformis* (Table 1), PFs generally exhibited higher mean sensitivity (MS), standard deviation (SD) of ring width, and signal-to-noise ratio (SNR) compared with NFs at most sampling sites (e.g., BH, MW, HL, and QY). This finding indicates that PF trees are more sensitive to environmental changes, with greater interannual variability and stronger climatic signals observed in their radial growth. In addition, NFs showed higher inter-series correlation coefficients (Rbt) than PFs at certain sites (e.g., MW, HL, QY, and BJ), which reflects a stronger growth synchrony among trees under natural environmental conditions.

### 2.2. Growth–Climate Correlations

**Figure 1:**
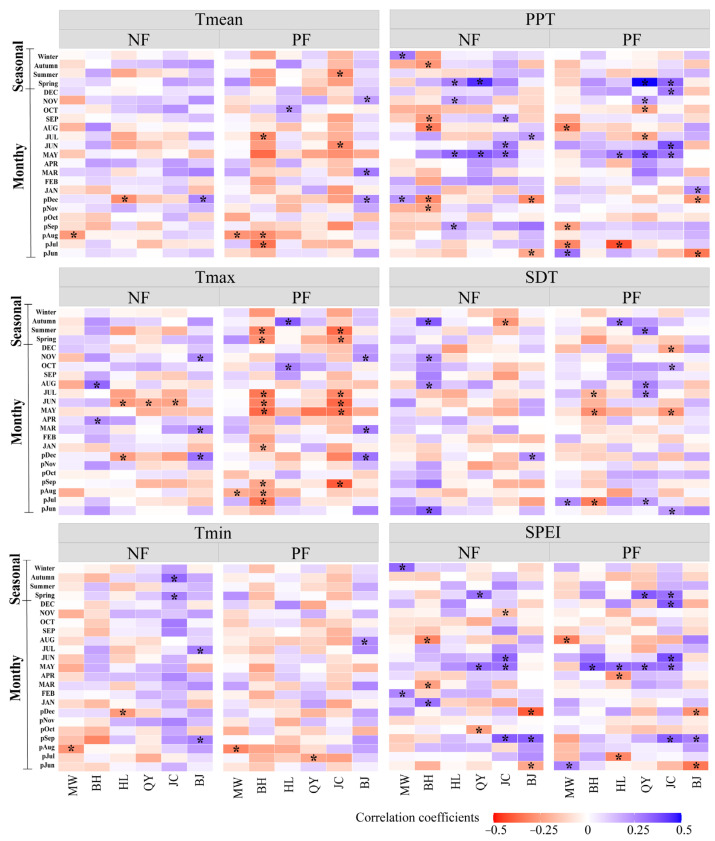
Correlation between the standard chronologies of NFs and PFs and climate variables (monthly and seasonal). Note: “*” indicates significant correlation (*p* < 0.05). The previous year’s months are indicated by “p” (e.g., pJul represents July of the previous year). Tmean: mean temperature; Tmax: maximum temperature; Tmin: minimum temperature; PPT: total precipitation; SDT: total sunshine duration; SPEI: standardized precipitation evapotranspiration index. MW: Muwang site; BH: Baihua site; HL: Huanglong site; QY: Qinyuan site; JC: Jiaocheng site; BJ: Beijing site.

The correlations between the chronologies of NFs and PFs with climatic variables (monthly and seasonal) revealed a considerably stronger climate sensitivity in PFs compared with NFs (Figure 1). Both forest types exhibited significant negative correlations between radial growth and Tmean from August of the previous year to June of the current year. However, NFs demonstrated a generally lower temperature sensitivity, with notable associations limited to specific regions. The Tmean and Tmin in August of the previous year showed significant negative correlations with growth in the MW region, and June Tmax displayed significant negative correlations in the HL, QY, and JC regions. Notably, northern sites (e.g., BJ region) displayed significant positive correlations between growth and Tmean/Tmax from December of the previous year. By contrast, PF growth exhibited a stronger temperature sensitivity with more pronounced thermal constraints. Specifically, significant negative correlations with Tmean/Tmax occurred during July–August of the previous year in the MW and BH regions, and during May–July in the BH and JC regions. However, northern sites (e.g., BJ) revealed positive correlations with December (previous year), March, and November (current year) Tmean/Tmax.

Furthermore, September SPEI of the previous year and May PPT/SPEI of the current year showed a positive correlation with radial growth in both forest types (Figure 1). Compared with NFs, PF growth demonstrated greater susceptibility to PPT, SPEI, and SDT influences: July PPT of the previous year exerted stronger inhibitory effects on PFs in the MW and HL regions; May–June SPEI enhanced PF growth more substantially than in NFs in the BH, HL, QY, and JC regions; and May SDT imposed greater negative impacts on PFs in the BH and JC regions.

### 2.3. Temporal Stability of Growth–Climate Relationships

**Figure 2:**
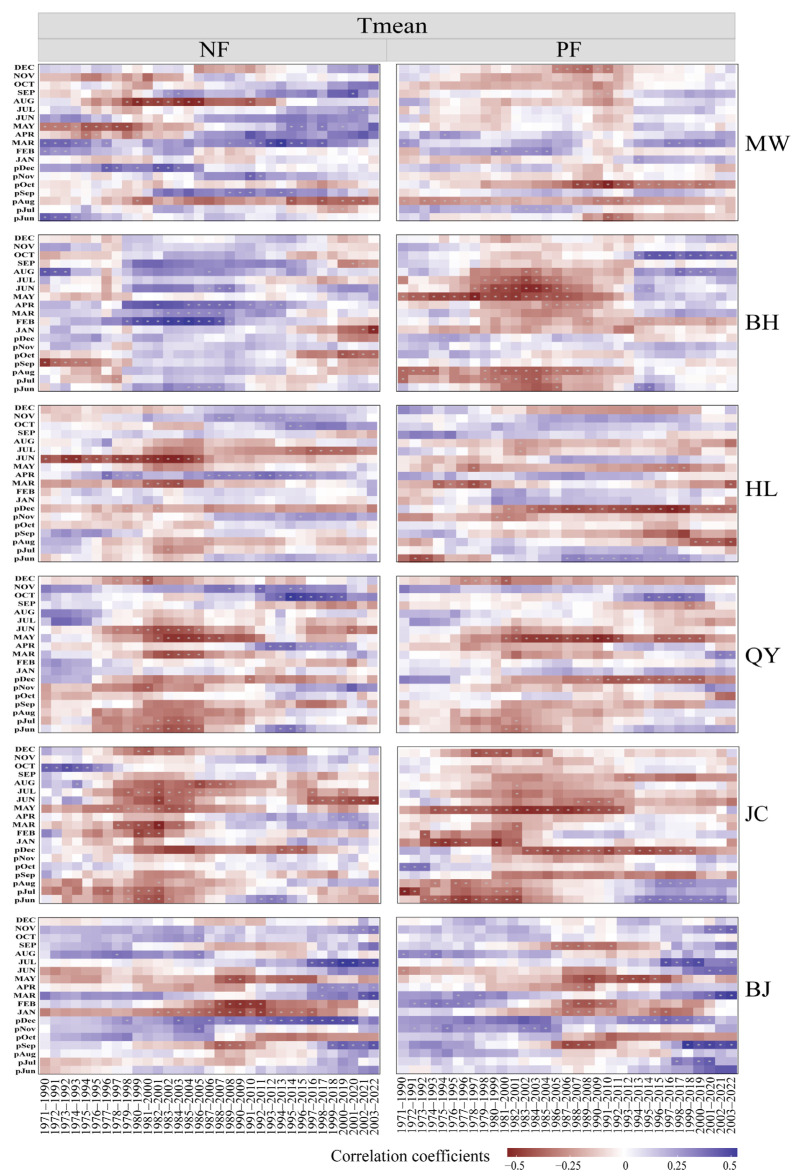
Moving correlation analysis (20-year window) between tree-ring width of *P. tabulaeformis* in NFs and PFs and monthly temperature. Note: The previous year’s months are indicated by “p” (e.g., pJul represents July of the previous year). Tmean: mean temperature. MW: Muwang site; BH: Baihua site; HL: Huanglong site; QY: Qinyuan site; JC: Jiaocheng site; BJ: Beijing site.

**Figure 3:**
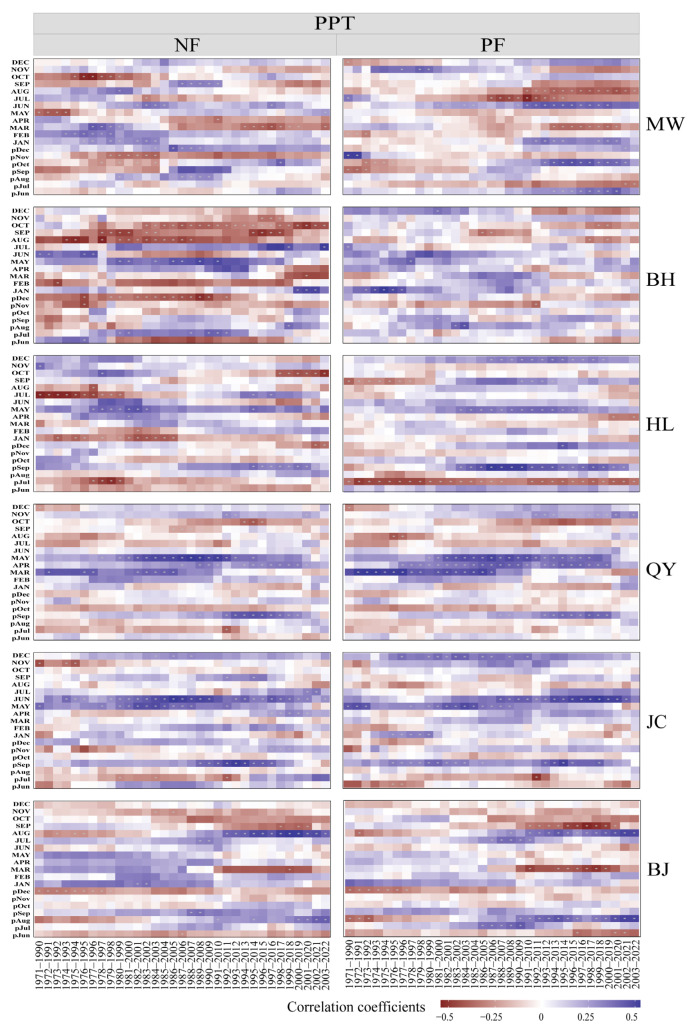
Moving correlation analysis (20-year window) between tree-ring width of *P. tabulaeformis* in NFs and PFs and monthly precipitation. Note: The previous year’s months are indicated by “p” (e.g., pJul represents July of the previous year). PPT: total precipitation. MW: Muwang site; BH: Baihua site; HL: Huanglong site; QY: Qinyuan site; JC: Jiaocheng site; BJ: Beijing site.

The temporal stability analysis of radial growth responses to climate variability in NFs and PFs revealed marked differences in their sensitivities to temperature and precipitation (Figure 2 and Figure 3). Plantations exhibited a higher sensitivity to climatic fluctuations, with growth–climate correlations showing a more pronounced temporal variability, whereas NFs maintained relatively stable response patterns in certain periods and regions (Figure 2 and Figure 3).

Specifically, NFs displayed stable temperature responses in specific months. Positive correlations persisted between growth and March temperatures in the MW region, April temperatures in HL, and December (previous year) temperatures in BJ. However, negative correlations with August (previous year) temperatures in MW and June–July temperatures in HL implied remarkable strengthening or weakening trends over time (Figure 2). For precipitation responses, stable positive correlations were observed with May precipitation in BH, September (previous year) precipitation in HL, and June precipitation in JC. Meanwhile, stable negative correlations occurred with November (previous year) precipitation in MW and August–October/December precipitation in BH (Figure 3). Notably, significant shifts in temperature and precipitation correlations transpired around 1980–1999 and 1990–2009 in some regions.

In PFs, temperature responses showed stable negative correlations with August (previous year) temperatures in MW and May temperatures in QY. However, most regions experienced shifts in correlations around 1990–2009 (Figure 2). HL transitioned from negative to positive correlations with June (previous year) temperatures and JC from negative to positive correlations with June–August (previous year) temperatures (Figure 2). Stable precipitation responses were observed in April–May correlations in QY and September (previous year) correlations in JC. Nevertheless, precipitation correlations in the MW, JC, and HL regions underwent notable changes around 1990–2009 (Figure 3).

### 2.4. Spatial Gradient Analysis of Growing-Season Variables

**Figure 4:**
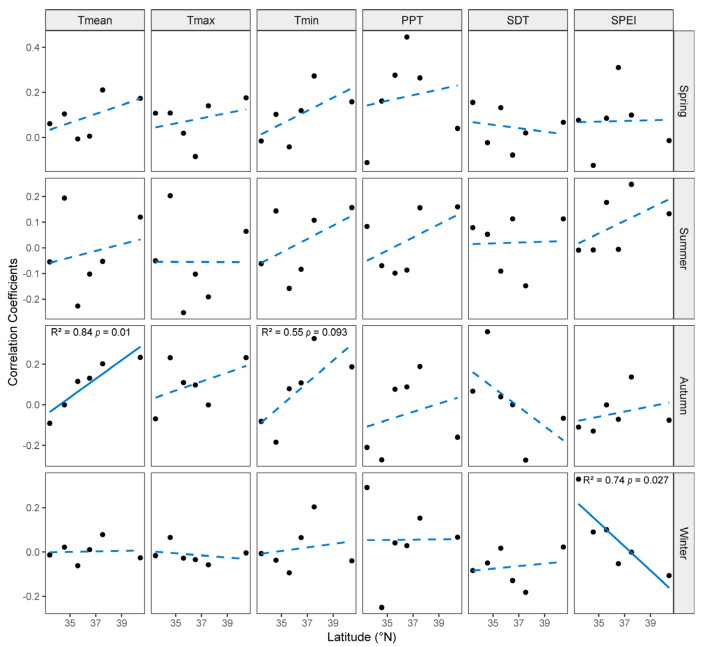
Relationships between radial growth–climate correlations and latitude in NFs. Note: In each panel, fitted lines from simple linear regression are displayed as solid lines for *p* ≤ 0.05 and dashed lines for *p* > 0.05. The coefficient of determination (R^2^) is reported for relationships with *p* ≤ 0.1. Tmean: mean temperature; Tmax: maximum temperature; Tmin: minimum temperature; PPT: total precipitation; SDT: total sunshine duration; SPEI: standardized precipitation evapotranspiration index.

The growth–climate relationships of NFs and PFs exhibited significant spatial divergence (Figure 4). In NFs, correlations between radial growth and autumn Tmean increased linearly with latitude, transitioning from negative to positive associations, whereas correlations with winter SPEI decreased linearly with latitude, with a positive-to-negative shift being observed. By contrast, PFs showed no significant latitudinal trends in growth–climate correlations (Figure S3).

### 2.5. Relative Importance of Climatic Variables in NFs and PFs

**Figure 5:**
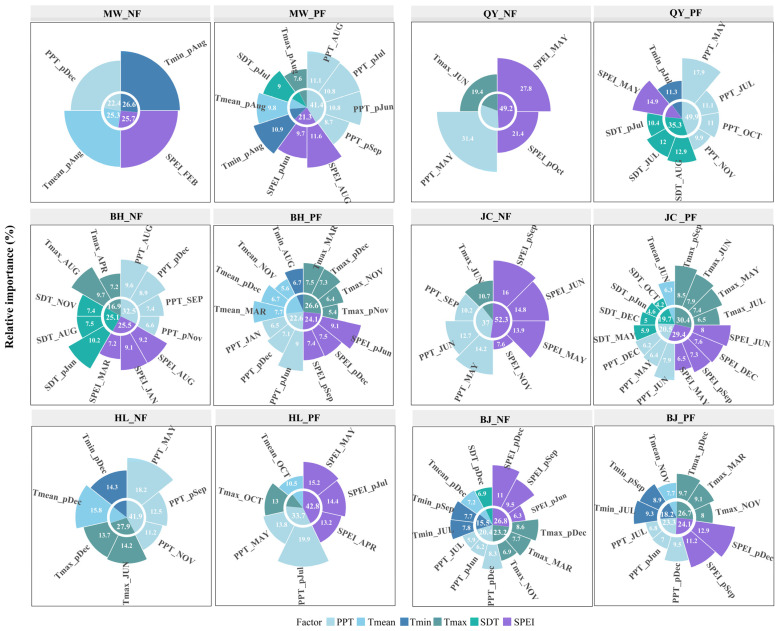
Relative importance of different variables in explaining variation in the radial growth of NFs and PFs at different sites. Note: The previous year’s months are indicated by “p” (e.g., pJul represents July of the previous year). Tmean: mean temperature; Tmax: maximum temperature; Tmin: minimum temperature; PPT: total precipitation; SDT: total sunshine duration; SPEI: standardized precipitation evapotranspiration index. MW: Muwang site; BH: Baihua site; HL: Huanglong site; QY: Qinyuan site; JC: Jiaocheng site; BJ: Beijing site.

Climate variables were assessed for their relative importance in explaining the variation in radial growth across sampling sites (Figure 5 and Figure S4). At the regional scale, PPT and SPEI exhibited higher relative importance for NFs (30.9% and 29.9%, respectively). For PFs, Tmax (21.4%) and SDT (13.1%) ranked second to PPT and SPEI (27% and 23%, respectively). Overall, hydrothermal balance (PPT and SPEI) predominantly governs NF growth, whereas multiple factors (PPT, SPEI, Tmax, and SDT) impact the integrated regulation of PF growth, which indicates heightened sensitivity and systemic complexity in PFs’ response to climate change.

## 3. Discussion

### 3.1. Relationships Between Radial Growth and Climatic Factors

Significant negative correlations were observed between radial growth in NFs and PFs and the Tmean of August in the previous year and the Tmax of June in the current year. This finding indicates that elevated summer temperatures suppressed *P. tabulaeformis* growth with a lagged effect, consistent with prior studies [35,36,37]. Elevated summer temperatures likely enhance plant transpiration, reduce soil water availability, induce partial stomatal closure, and decrease photosynthetic rates, limiting organic synthesis and carbon storage [36,38,39]. Concurrently, elevated summer temperatures often trigger drought events, which cause xylem embolism, impair hydraulic conductivity, and further inhibit growth [40,41]. In addition, elevated temperatures in the previous summer may reduce carbon reserves via increased respiratory costs and water stress, which diminish energy and material reserves for subsequent xylem formation [42,43,44]. Radial growth in both forest types showed significant positive correlations with May PPT and SPEI during the current year, which suggests that ample early-growing-season moisture promotes growth; this outcome aligns with previous findings [45,46,47]. Increased precipitation at the onset of the growing season increases soil moisture, which facilitates cambial activity and earlywood formation [48,49,50,51].

Divergent growth–climate responses between NFs and PFs have been widely reported [26,52,53], with most studies reporting a higher climatic sensitivity in plantations [54,55,56]. This study found that tree radial growth in PFs was more sensitive to climate variability than that in NFs. Specifically, the temperatures in both the previous and current growing seasons, along with SPEI during the current growing season, had significantly stronger inhibitory effects on tree growth in PFs. Camarero et al. [26] suggested that PFs generally have lower species diversity and structural complexity, making them more vulnerable to climate change. Similarly, Sánchez-Salguero et al. [57] and Yu et al. [56] emphasized that the homogeneous structure of PF could reduce their adaptive capacity. However, Ni et al. [28] reported largely similar temperature effects on radial growth in NF and PF stands of *P. massoniana* in central subtropical regions. These divergent findings may result from differences in species distribution, ecological characteristics, and species-specific responses [58]. Notably, Ni et al. [28] also observed that SPEI had stronger explanatory power for radial growth in PFs than in NFs, which is consistent with the stronger influence of SPEI on PF growth identified in this study. PFs usually consist of a single tree species with low genetic diversity and a homogeneous stand structure, resulting in heightened sensitivity to climate fluctuations [59]. In diverse forest ecosystems, different species typically have varying vertical root distributions: shallow, intermediate, and deep roots coexist to collectively exploit soil moisture at different depths [60]. In contrast, PFs composed of a single species typically exhibit uniform rooting depths, making them more susceptible to drought. Furthermore, PFs generally originate from a limited number of provenances, resulting in lower genetic diversity. Genetic diversity has been positively correlated with environmental adaptability [61]. Empirical studies have also reported negative outcomes associated with pure stands of *P. tabuliformis*, such as limited understory species richness, accelerated decline in tree growth, and reduced resistance to natural disturbances [62,63]. Compared to PFs, NFs have more complex ecological structures and greater heterogeneity. NFs typically have multi-layered canopies and mixed-species compositions, facilitating complementary resource utilization. Diverse canopy structures and varied root systems among different tree species enable the stratified exploitation of resources such as light, water, and nutrients, thereby enhancing overall ecosystem stability [64]. Moving window analyses revealed temporal shifts in growth–climate relationships, particularly during 1980–1999 and 1990–2009; these were likely driven by accelerated global warming, rising vapor pressure deficits (VPDs), and intensified regional hydroclimate variability [38,65,66]. For example, Li et al. [67] indicated that during drought years, when soil water availability decreases, tree growth becomes more dependent on precipitation and the adverse impact of high temperature on tree growth intensifies. Jia et al. [16] found that the radial growth of Larix principis-rupprechtii in NFs in Northern China was not significantly affected by water stress before an abrupt temperature shift; however, after this temperature shift, water stress became a limiting factor for radial growth. PFs exhibited higher temporal variability in growth–climate relationships than NFs did, reflecting PFs’ vulnerability to climatic extremes under monoculture management. This may be attributed to the higher stand density and more homogeneous growing conditions in PFs, which make PFs more susceptible to climatic anomalies [68,69]. Similarly, Jia et al. [16] reported that after an abrupt temperature shift, structurally homogeneous PFs of L. principis-rupprechtii experienced greater declines in radial growth due to water stress, whereas structurally complex NFs exhibited higher stability and stronger adaptive capacity to climatic fluctuations. These findings underscore the phase-dependent effects of climate change on forest growth and highlight critical differences in the response stability of NFs and PFs, which necessitate tailored management strategies.

### 3.2. Spatial Shifts in Climate–Growth Relationships

The correlations between climatic factors and radial growth varied across sampling sites and forest types. In NFs, autumn Tmean revealed an inhibitory-to-facilitative influence on radial growth along the south-to-north latitudinal gradient. This finding is consistent with Ni et al. [28], who reported that with increasing latitude, the influence of summer temperature on the growth of *P. massoniana* in PFs shifts from inhibitory to promotive. In warmer southern regions, elevated autumn temperatures likely induced drought stress, which suppresses radial growth [70]. Conversely, in northern regions, elevated autumn temperatures extended the growing season, which enhanced photosynthetic duration and biomass accumulation [71,72]. Furthermore, NF radial growth and winter SPEI showed a correlation shift from positive in the south to negative in the north, which indicates the weakening of drought limitation with increases in latitude. In southern areas, humid winters replenished soil moisture reserves, which benefits subsequent-year growth. By contrast, excessive winter moisture in northern regions may impair growth via frost damage or root waterlogging stress [20,73,74]. Thus, under sustained global warming, climate change may reshape regional growth response patterns in NFs, with northern *P. tabulaeformis* likely exhibiting enhanced radial growth and southern populations facing drought-driven declines [75,76].

By contrast, PFs exhibited no clear latitudinal trends in climate–growth relationships. This finding is primarily attributed to the dominant role of anthropogenic management—such as species selection, irrigation, fertilization, and density control—in overriding natural climatic gradients [77,78,79]. PFs often employ genetically uniform, climate-adapted provenances alongside intensive silvicultural interventions, which buffer latitudinal climatic constraints [80,81]. These findings underscore the necessity of integrating forest-type-specific responses into predictive models of climate change impacts and adaptive management frameworks, particularly when designing regionally tailored conservation strategies [75].

### 3.3. Relative Importance of Climatic Factors

Growing evidence suggests that tree growth worldwide is increasingly constrained by atmospheric water demand [82]. Our study revealed that at regional scales, PPT and SPEI exerted stronger influences on radial growth in NFs and PFs than temperature (Figure 5 and Figure S3). This finding is in contrast with that of Huang et al. [83] and Ni et al. [28], who identified temperature as the dominant driver of *P. massoniana* growth. Such discrepancies possibly reflect regional climatic and forest-type differences. Huang and Ni’s studies focused on warm–humid Southern China, where abundant moisture minimizes hydrological constraints, which amplifies temperature effects [84,85]. By contrast, our northern study area experiences frequent droughts, and water availability (PPT and SPEI) directly governs photosynthesis, transpiration, and water-use efficiency [45,86]. Global warming is projected to exacerbate soil moisture deficits via rising VPDs, which further intensify precipitation dependencies [26,78]. Consequently, northern forests exhibit a stronger growth reliance on moisture inputs, which highlights the regional and ecosystem-specific nature of climatic drivers.

PPT and SPEI explained most radial growth variations in NFs, which is in alignment with the findings of Camarero et al. [26], who underscored water limitation as a critical constraint. In PFs, however, Tmax and SDT, which are likely linked to PFs’ ecological traits and management regimes, emerged as secondary yet notable drivers [87]. These findings suggest that NF growth is primarily hydroclimatically regulated, whereas PFs are influenced by multifactorial climatic interplay. NF conservation should prioritize water optimization (e.g., thinning and litter layer management), and PF management requires multidimensional strategies (e.g., shading, density control, and deep-rooted genotype selection) to buffer compound climatic stresses.

## 4. Materials and Methods

### 4.1. Study Sites

The study area is located within the typical distribution zone of *P. tabulaeformis* in the warm, temperate region of Northern China, and encompasses Gansu, Shaanxi, and Shanxi provinces and the Beijing municipality (Figure S1). Six sites were included, spanning a latitudinal range of 8 degrees (33–41° N). Each site comprises mature NF and PF. The selected NFs derive from natural regeneration and display a long-term absence of human intervention and dense understory vegetation. By contrast, PFs were established through national or local forestry programs. Trees in NFs have an average tree age of 82 ± 13 years, whereas those in PFs have an average tree age of 59 ± 11 years.

### 4.2. Sample Processing and Chronology Development

Field sampling was conducted during the growing seasons of 2021–2022. At each study site, 3–4 plots (20 m × 30 m each) were established for NFs and PFs, with a minimum distance of 1 km maintained between plots of the same forest type. Within each plot, five healthy dominant trees with similar diameters at breast height were selected. Two tree cores per tree were extracted at 1.30 m above ground using a 5.15 mm diameter increment borer, following a vertical direction.

In the laboratory, the cores were air-dried naturally, glued to wooden mounts, and secured with clamps to prevent deformation. After drying, the cores were sanded using progressively finer grits of sandpaper until annual ring boundaries became visible. Visual cross-dating was performed on each sample, and ring widths were measured on a LINTAB ring width measurement system (Rinntech, Heidelberg, Germany) to an accuracy of 0.01 mm. The accuracy of cross-dating was verified using the COFECHA v.6.06P [88]. Samples with low COFECHA correlation coefficients were excluded from subsequent analyses. Standard chronologies for each sampling site were developed using the “dplR” package in R (Figure S2). To characterize chronology quality, we calculated dendrochronological statistical metrics, including the mean inter-series correlation coefficient (Rbar), expressed population signal (EPS), and signal-to-noise ratio (SNR) (Table 1).

### 4.3. Climate Variables

**Figure 6:**
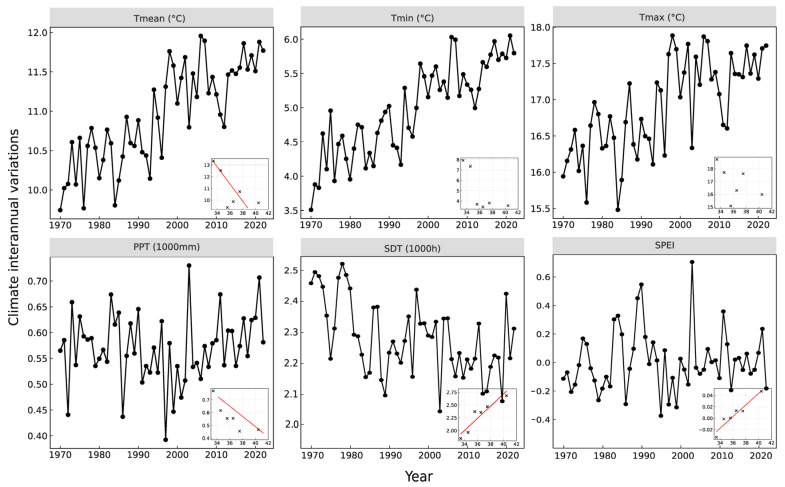
Interannual climate variability during the common period (1970–2022) and bivariate relationships between latitude and annual mean climate variables (shown in each subplot). Note: In each subplot, a simple linear regression line is plotted when *p* < 0.05. Tmean: mean temperature; Tmax: maximum temperature; Tmin: minimum temperature; PPT: total precipitation; SDT: total sunshine duration; SPEI: standardized precipitation evapotranspiration index.

All climate variables used in this study were recorded on a monthly scale, including mean monthly temperature (Tmean, °C), minimum temperature (Tmin, °C), maximum temperature (Tmax, °C), monthly precipitation (PPT, mm), and the standardized precipitation evapotranspiration index (SPEI). This information was obtained from the Climate Explorer website (http://climexp.knmi.nl, accessed on 1 March 2024). Sunshine duration (SDT, h) data were extracted from meteorological station records in Southern China (China Meteorological Data Service Center, https://data.cma.cn, accessed on 1 May 2024) and spatially interpolated using the ordinary Kriging method in ArcGIS 10.0 (Environmental Systems Research Institute, Redlzands, CA, USA). Based on these datasets, during 1970–2022, the annual mean temperature (Tmean), minimum temperature (Tmin), and maximum temperature (Tmax) were in the range of 9.74–11.88 °C, 3.51–6.31 °C, and 15.58–17.87 °C, respectively. Annual precipitation (PPT) varied between 392.32 and 730.05 mm, annual SDT between 2045.75 and 2521.99 h, and SPEI between −0.317 and 0.703 (Figure 6).

### 4.4. Data Processing

This study involved the analysis of the climate–growth relationships of NFs and PFs along a climatic gradient. Correlation analyses (Pearson correlation coefficients) were conducted to assess the associations between the chronologies of NFs and PFs and monthly/seasonal climate variables (Tmean, Tmax, Tmin, PPT, SDT, and SPEI). To further investigate the dynamic impacts of climatic factors, we performed a moving window correlation analysis (20-year window) to evaluate the temporal stability of the relationships between tree-ring width indices and climate variables. Stepwise regression analysis was conducted to quantify the relative importance of climatic variables (expressed as the percentage of variance explained) on radial tree growth across sampling sites. All figures in this study were generated using R 4.3.1 (R Core Team, Vienna, Austria), Origin 9.0 (OriginLab, Northampton, MA, USA), and ArcGIS 10.0 (Environmental Systems Research Institute, Redlzands, CA, USA).

## 5. Conclusions

Comprehending divergent climatic responses between NF and PF is critical for the development of science-based forest management strategies under climate change. Our results demonstrate the higher sensitivity of PFs to climatic factors and greater temporal variability in growth–climate relationships compared with NFs. In addition, the relative importance of climatic drivers differed by forest type: NF growth was predominantly governed by water availability (PPT and SPEI), whereas PF growth was co-regulated by interactive temperature and moisture effects. Spatial analyses revealed latitudinal divergence in NF growth–climate linkages, with growth-inhibitory autumn temperatures in southern regions shifting to growth-promoting ones in northern regions and winter moisture transitioning from facilitative to suppressive along the same gradient. By contrast, PFs showed no significant latitudinal trends, which reflects the human-mediated homogenization of climatic constraints. These findings highlight how forest origin shapes tree’s climate adaptation strategies and provide a theoretical foundation for tailored conservation (e.g., hydrological optimization in NFs) and adaptive silviculture (e.g., multifactorial stress mitigation in PFs) in a warming world.

## Figures and Tables

**Table 1:** Sampling site information and standard chronology statistical characteristics.

Indicators	BH		MW		HL		JC		QY		BJ	
	NF	PF	NF	PF	NF	PF	NF	PF	NF	PF	NF	PF
Time span	1950–2022	1950–2022	1920–2022	1975–2022	1930–2022	1975–2022	1937–2022	1967–2022	1952–2022	1969–2022	1954–2024	1970–2022
MD	0.942	0.962	0.978	0.944	0.953	0.980	0.985	0.988	0.963	0.986	0.986	0.976
MS	0.166	0.170	0.096	0.163	0.132	0.141	0.118	0.167	0.188	0.148	0.241	0.178
SD	0.190	0.197	0.131	0.202	0.155	0.169	0.146	0.190	0.198	0.155	0.259	0.228
AC1	0.587	0.417	0.558	0.457	0.439	0.506	0.498	0.328	0.302	0.201	0.186	0.544
Rbt	0.422	0.556	0.438	0.399	0.520	0.397	0.424	0.544	0.498	0.496	0.624	0.524
SNR	7.582	18.881	6.584	6.764	7.786	7.795	7.263	9.542	10.081	9.409	18.067	13.246
EPS	0.883	0.95	0.868	0.871	0.921	0.872	0.924	0.905	0.91	0.904	0.966	0.93

Note: NF: natural forest; PF: planted forest; MD: mean; MS: mean sensitivity; SD: standard deviation; AC1: first-order autocorrelation; Rbt: mean inter-series correlation; SNR: signal-to-noise ratio; EPS: expressed population signal. MW: Muwang site; BH: Baihua site; HL: Huanglong site; QY: Qinyuan site; JC: Jiaocheng site; BJ: Beijing site.

## Data Availability

The data presented in this study are available on request from the corresponding author.

## Supplementary Materials

The following supporting information can be downloaded at: https://www.mdpi.com/article/10.3390/plants14101441/s1, Figure S1: Location map of sampling plots; Figure S2: Chronology of tree-ring widths for NF and PF of *P. tabulaeformis* at different sites; Figure S3: Relationships between radial growth-climate correlations and latitude in PF; Figure S4: Average relative importance of climatic variables in explaining radial growth variations in NF and PF.
