# Supplementary material for: Divergent Climate Sensitivity and Spatiotemporal Instability in Radial Growth of Natural and Planted Pinus tabulaeformis Forests Across a Latitudinal Gradient
Source: Plants (Basel). 2025 May 12;14(10):1441. doi: 10.3390/plants14101441 (PMC12115013; doi:10.3390/plants14101441)
Supplement: Supplementary file 1 [file plants-14-01441-s001.zip › plants-3586223-supplementary.pdf]

Additional Figures:

Figure S1. Location map of sampling plots.

Figure S2. Chronology of tree-ring widths for NF and PF of *P. tabulaeformis* at different sites.

Figure S3. Relationships between radial growth-climate correlations and latitude in PF.

Figure S4. Average relative importance of climatic variables in explaining radial growth variations in NF and PF.

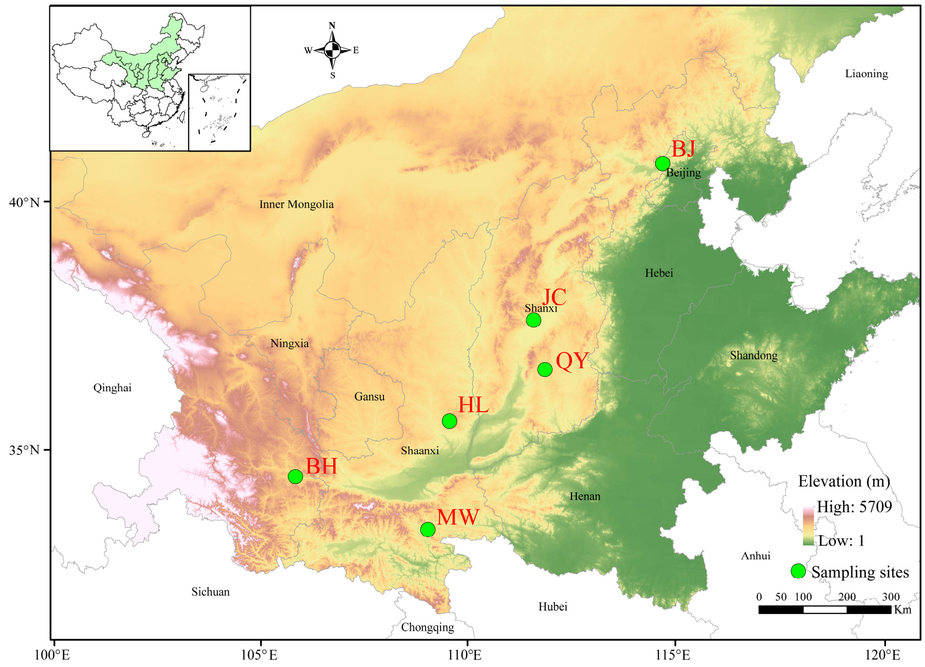

Figure S1. Location map of sampling plots. Note: MW: Muwang site; BH: Baihua site; HL: Huanglong site; QY: Qinyuan site; JC: Jiaocheng site; BJ: Beijing site.

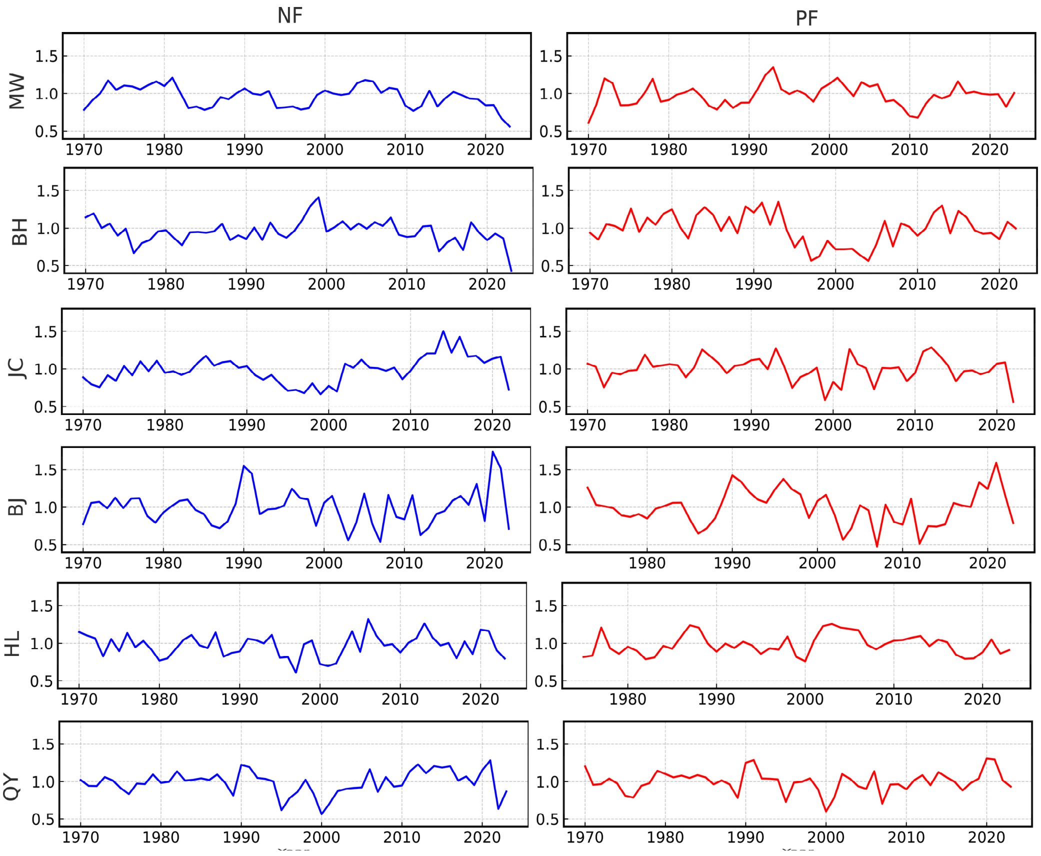

Figure S2. Chronology of tree-ring widths for NF and PF of *P. tabulaeformis* at different sites. Note: MW: Muwang site; BH: Baihua site; HL: Huanglong site; QY: Qinyuan site; JC: Jiaocheng site; BJ: Beijing site.

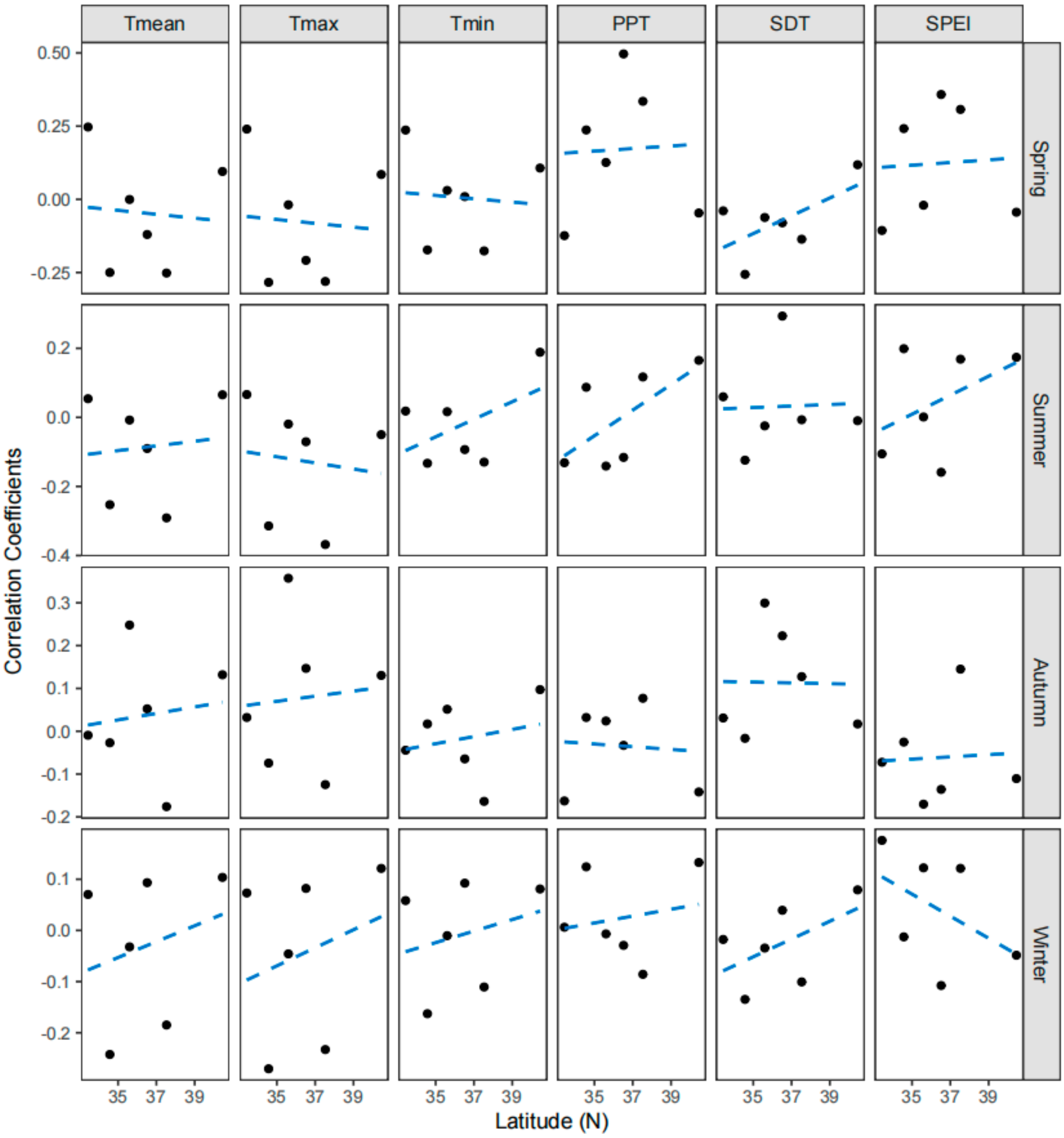

Figure S3. Relationships between radial growth-climate correlations and latitude in PF. Note: In each panel, fitted lines from simple linear regression are displayed as solid lines for $p \leq 0.05$ and dashed lines for $p > 0.05$. The coefficient of determination (R^2) is reported for relationships with $p \leq 0.1$. Tmean: mean temperature; Tmax: maximum temperature; Tmin: minimum temperature; PPT: total precipitation; SDT: total sunshine duration; SPEI: standardized precipitation evapotranspiration index.

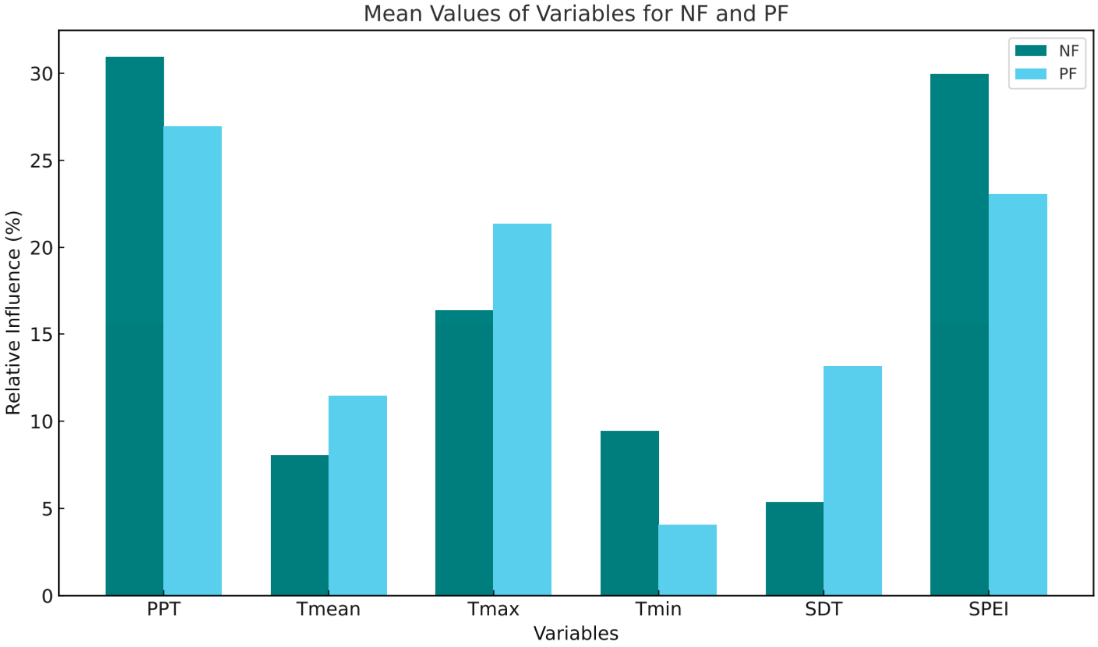

Figure S4. Average relative importance of climatic variables in explaining radial growth variations in NF and PF. Note: Tmean: mean temperature; Tmax: maximum temperature; Tmin: minimum temperature; PPT: total precipitation; SDT: total sunshine duration; SPEI: standardized precipitation evapotranspiration index.
